# Artificial intelligence-driven framework for improving prenatal screening for congenital heart disease in rural Nebraska

**DOI:** 10.3389/fped.2025.1653305

**Published:** 2025-10-08

**Authors:** Ling Li, Alex J. Foy, Jason T. Christensen, Aaron Lanik, Jieqiong Wang, Neil Hamill, Jeffrey W. Delaney, S. Ram Kumar

**Affiliations:** ^1^ Criss Heart Center, Children’s Nebraska, Omaha, NE, United States; ^2^ Department of Pediatrics, University of Nebraska College of Medicine, Omaha, NE, United States; ^3^ Department of Family Medicine, University of Nebraska College of Medicine, Omaha, NE, United States; ^4^ Department of Neurological Sciences, University of Nebraska College of Medicine, Omaha, NE, United States; ^5^ Department of Obstetrics and Gynecology, University of Nebraska College of Medicine, Omaha, NE, United States; ^6^ Department of Surgery, University of Nebraska College of Medicine, Omaha, NE, United States

**Keywords:** congenital heart disease (CHD), prenatal detection, healthcare disparities, artificial intelligence (AI), rural health access

## Abstract

**Purpose:**

Congenital heart disease (CHD) is the most common birth defect and a leading cause of neonatal morbidity and mortality. Despite advances in prenatal imaging, rural communities face persistent disparities in CHD detection due to limited access to specialized diagnostics. This position paper proposes an AI-enabled framework to embed early CHD detection into routine prenatal care and reduce the rural-urban gap in Nebraska.

**Method:**

A review of 1,502 surgical CHD cases at Children's Nebraska (2019–2024) revealed significant geographic disparities in prenatal detection. In response, we outline a framework that leverages a secure, cloud-based platform to apply AI algorithms to standard obstetric ultrasound images. Flagged cases are referred to nearby fetal cardiology outreach centers, reducing delays associated with centralized tertiary care access.

**Framework:**

This approach leverages existing infrastructure, including the Children's Nebraska fetal heart center, UNMC's rural residency network, and maternal-fetal medicine collaborations. Implementation will be led by an interdisciplinary team spanning cardiology, Obstetrics, rural health, imaging, and machine learning.

**Conclusion:**

By decentralizing diagnostics and enabling earlier triaging in community settings, this scalable, accessible framework offers a practical solution for improving prenatal CHD detection in underserved regions, with strong potential for national replication.

## Highlights

• Proposes a practical, AI-enabled framework to reduce prenatal CHD detection disparities.
• Highlights integration of machine learning into routine obstetric ultrasound workflows.
• Builds on existing rural health infrastructure for scalable, real-world implementation.
• Adds a systems-level perspective to AI in maternal-fetal care, beyond algorithm development.
• Establish a scalable, sustainable framework for AI-driven prenatal screening programs, with the potential to serve as a national model for improving maternal-fetal health outcomes.

## Introduction

Congenital Heart Disease (CHD) is the most prevalent birth defect worldwide, affecting approximately 40,000 births annually in the United States (1, 2). It remains the leading non-infectious cause of infant mortality, contributing significantly to neonatal deaths and maternal distress. CHD encompasses a broad spectrum of structural heart abnormalities, from mild defects requiring minimal intervention to critical congenital heart defects (CCHD), which demand urgent surgical or catheter-based intervention shortly after birth. The economic burden is substantial—hospital costs for individuals with heart defects exceeded $9.8 billion in 2019 (3), and when accounting for CCHD-related mortality, the total annual cost is estimated at $74 billion (4).

Despite advancements in medical imaging and prenatal care, detection rates for CHD remain inconsistent, averaging 50%–60% in well-resourced settings but falling below 30% in many rural areas (5). Even complex conditions like single ventricle defects are detected prenatally in only 75% of cases, with institutional detection rates ranging from 59% to 85% (6). Prior studies demonstrate prenatal diagnosis of CCHD enables earlier intensive care, reduces the risk of shock, and improves outcomes (7, 8). Improving prenatal detection, particularly of CCHD, is critical for enhancing neonatal survival and quality of care.

Early detection is especially crucial in underserved rural areas, where systemic healthcare disparities hinder access to timely diagnosis and specialized care. As the only comprehensive pediatric cardiac program in Nebraska, Criss Heart Center at Children's Nebraska serves a geographically expansive population with widely varying access to medical resources. As such, our overall objective is to reduce health disparities in prenatal CHD detection and management by integrating AI-driven technologies into population-level screening programs, thereby improving screening efforts in rural Nebraska and the Midwest. To that end, we chose to understand the scope of the problem of variable prenatal detection of CHD in the state. We propose leveraging Artificial Intelligence/Machine Learning (AI/ML) to enhance prenatal CHD detection through population-level screening programs, focusing on rural Nebraska and the broader Midwest region. The project's success will be driven by an interdisciplinary team with expertise spanning rural healthcare, pediatric and fetal cardiology, machine learning, and imaging science.

### Scope of the problem

Despite advances in prenatal imaging, disparities in CHD detection persist, particularly in rural communities. To evaluate these disparities, we conducted a retrospective analysis of all patients who underwent surgery to address CHD at Children's Nebraska from 2019 to 2024. Using the USDA Rural-Urban Continuum Codes, we defined geographic location as urban vs. rural. CHD was classified based on severity as CCHD or non-CCHD (NCCHD). Critical congenital heart disease (CCHD) refers to severe heart defects that necessitate surgical or catheter-based intervention within the first year of life to ensure survival and prevent significant morbidity or mortality (9). In contrast, non-critical CHD encompasses heart defects present that are not immediately life-threatening and typically do not require urgent intervention during the neonatal period. We examined prenatal vs. postnatal detection rates stratified by both geographic location and CHD severity.

**Figure 1:**
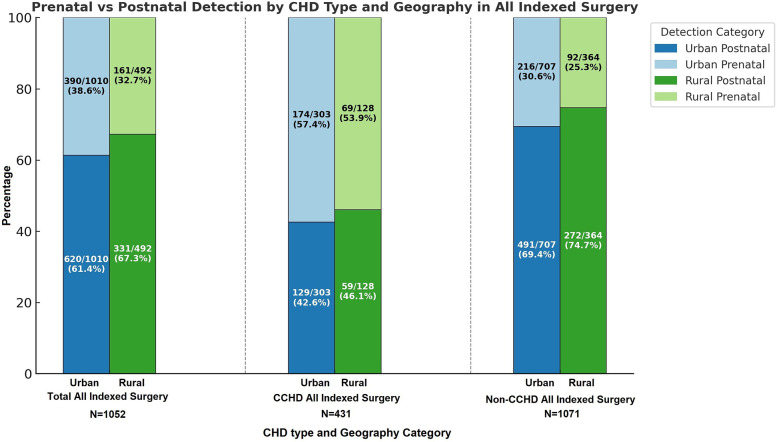
Percentage of defects detected pre- vs. post-natally in urban vs. rural areas in. Nebraska for both non-critical congenital heart disease (NCCHD) and critical congenital heart disease (CCHD) amongst patients who undergo surgery for congenital heart defects.

**Figure 2:**
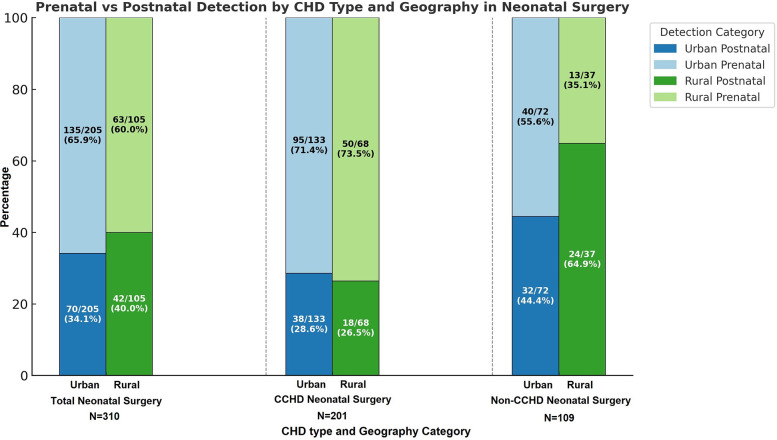
Percentage of defects detected pre- vs. post-natally in urban vs. rural areas in Nebraska for both non-critical congenital heart disease (NCCHD) and critical congenital heart disease (CCHD) amongst neonates (<28 days of age) who undergo surgery for congenital heart defects.

**Figure 3:**
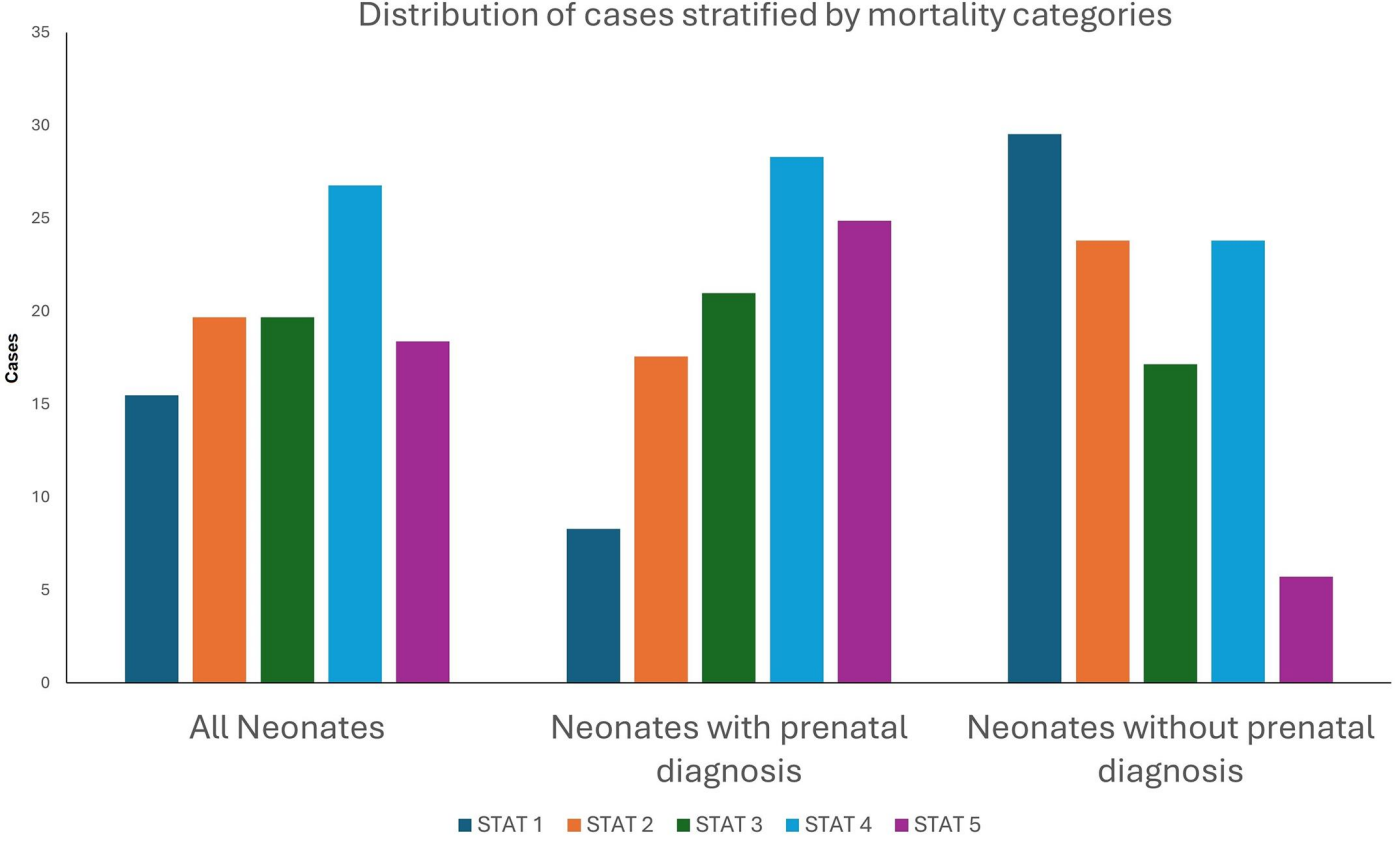
Percentage of defects detected pre- vs. post-natally amongst neonates (<28 days of age) who undergo surgery for congenital heart defects stratified by Society of Thoracic Surgeons—European Association for Cardio-Thoracic Surgery Congenital Heart Surgery (STAT) Mortality Risk Category (1–5), Stratified by Prenatal Diagnosis Status.

Among the 1,502 index cardiothoracic surgeries, 492 (32.76%) patients came from rural zip codes. Amongst these 1,502 cases, only 551 (36.68%) were detected prenatally, with higher detection rates in urban areas (390/1,010, 38.61%) compared to rural areas (161/492, 32.7%, *p* = 0.03). Postnatal diagnosis remained the predominant mode of detection, especially for non-CCHD (763/1,071, 71.24%), highlighting ongoing gaps in routine prenatal screening. There was a strong trend towards higher prenatal diagnosis in non-CCHD in the urban population (216/707, 30.55%) compared to the rural population (92/364, 25.27%, *p* = 0.07, Figure 1). In contrast, CCHD cases were more likely to be identified prenatally (243/431, 56.38%). Although prenatal detection rates of CCHD were higher in urban (174/303, 57.43%) compared to rural (69/128, 53.91%) populations, this difference was not statistically significant (*p* = 0.5) (Figure 1). We then looked at neonates requiring cardiothoracic surgery within the first 28 days of life (*n* = 310), who constitute a significantly higher-risk cohort of patients. The overall distribution of disease severity (non-CCHD vs. CCHD) did not significantly differ between urban (133/205 CCHD, 64.88%) and rural (68/105 CCHD, 64.76%, *p* = 0.98) groups. Urban neonates (135/205, 65.85%) demonstrated slightly, but not statistically significantly, higher prenatal detection rates compared to rural neonates (63/105, 60%, *p* = 0.31) across CHD severity. Given that the proportion of CCHD was higher among neonates compared to the overall cohort, the higher prenatal detection rate is congruent with data from our overall cohort analysis detailed above. In contrast to the overall population, prenatal detection rates of CCHD were slightly higher in rural (50/68, 73.53%) compared to urban (95/133, 71.43%) neonatal populations, but this difference was not statistically significant (*p* = 0.75). In contrast, there was a statistically significantly higher prenatal diagnosis rate in non-CCHD in the urban neonatal population (40/72, 55.55%) compared to the rural population (13/37, 35.14%, *p* = 0.04, Figure 2). Antenatally diagnosed neonates were significantly more likely to require higher mortality risk STAT 4 and STAT 5 procedures compared to those diagnosed postnatally (*p* = 0.0014). Gaps remain in the prenatal diagnosis of lower mortality risk procedures, where milder but surgically significant CHD lesions are often missed during fetal screening. Nearly two-thirds (31/48, 64.6%) of neonates requiring STAT 1 procedures were postnatally diagnosed, as were almost half (25/61, 41.0%) of those requiring STAT 2 procedures. In contrast, less than one-third (18/61, 29.5%) of STAT 3 cases and 25/83 (30.1%) of STAT 4 cases were diagnosed postnatally. Only 10.5% (6/57) of STAT 5 cases were missed prenatally, suggesting stronger fetal screening performance for high-risk conditions (Figure 3). These data highlight a significant disparity in the ability to diagnose lower-risk CCHD prenatally, likely due to subtler anatomic abnormalities that are more challenging to detect during fetal screening. For example, coarctation of the aorta (requiring STAT 1 procedure for repair) is inherently more challenging to identify prenatally compared to hypoplastic left heart syndrome (requiring STAT 5 procedure for palliation), which presents with more overt structural abnormalities on fetal echocardiography. The improvement in prenatal detection of complex CHD is encouraging, yet the persistent gap in diagnosing lower-mortality lesions remains a significant concern. Specifically, the median time-to-surgery for patients with prenatal detection was 7 days (IQR: 5–10 days), compared to 8 days (IQR: 5–14 days) for those with postnatal detection. This difference was statistically significant (*p* = 0.017, Mann–Whitney *U*-test), reinforcing the clinical importance of early diagnosis.

Based on these data, we define the scope of our problem with prenatal detection in the following areas –

• Amongst neonates requiring surgical intervention for CHD, current contemporary prenatal detection rates are only 63.9%. The absence of early diagnosis poses substantial challenges for postnatal stabilization and timely surgical planning, as highlighted by evidence demonstrating increased perioperative risk when CHD is identified only after birth (7). Moreover, prenatal diagnosis of CHD has been associated with improved long-term neurocognitive outcomes, likely due to optimized perinatal management and early intervention strategies (10). Early diagnosis also offers families the opportunity to mentally and logistically prepare for the complex care journey ahead—providing a sense of order and agency in what might otherwise be a chaotic and overwhelming postnatal experience (8).
• The need for an appropriate birth plan has significant importance in the child with CCHD. This is particularly relevant in a geographically expansive state like Nebraska, where Children's Nebraska serves as the sole pediatric cardiac care center. In such a setting, the value of prenatal planning cannot be overstated. To that end, 86% (43/50) of rural patients who had a fetal diagnosis of CCHD moved their delivery center to Omaha closer to Children's Nebraska. The 27% (18/68) rural patients who were not prenatally diagnosed were disadvantaged given their inability to exercise this choice of delivering their child closer to the center that can provide the requisite stabilizing care for the CCHD immediately after birth. This can result in a less stable patient going into surgery and accounts for the delay in time to surgery in these patients.
• Nearly 2/3rds of non-CCHD in rural neonates being diagnosed postnatally has very serious negative consequences. Not all non-CCHD is diagnosed prior to discharge of these neonates after birth. These defects may present with subtler signs early and may not become apparent until the child is in extremis. Many of these children do not have immediate access to medical care and transport facilities once discharged from the birthing center. As such, lack of prenatal diagnosis of non-CCHD poses a substantially disproportionate therapeutic burden on rural neonates.
• At our institution in Nebraska, only a little more than one-third of all CHD cases are diagnosed prenatally in the contemporary era presents an important opportunity to improve delivery of care across urban and rural populations.

For all these reasons, the lack of prenatal diagnosis exacerbates healthcare disparities, disproportionately affecting rural families, who often face additional logistical, financial, and access-related barriers in obtaining specialized pediatric cardiac care. These inequities underscore the urgent need for expanded access to advanced diagnostic technologies that can be delivered through existing infrastructure, particularly in underserved and remote regions. To address the challenges in diagnosing more subtle, yet clinically meaningful, forms of CHD, we propose a solution that integrates AI-driven prenatal CHD screening into existing healthcare workflows. By enhancing the sensitivity and reach of fetal cardiac evaluation, particularly in rural communities, this approach holds promise for ensuring timely diagnosis and improving outcomes for all patients, regardless of geographic location.

### Foundation and rationale for AI-driven screening

AI, particularly machine learning (ML), has shown transformative potential in improving prenatal CHD detection by augmenting fetal ultrasound interpretation and enhancing diagnostic consistency. Multiple studies have demonstrated high sensitivity and reproducibility of AI models in identifying structural heart abnormalities, particularly when applied to standardized ultrasound imaging inputs such as axial sweep cine loops (11–13). These inputs are commonly acquired during second-trimester anatomy scans and are routinely collected even in rural settings. In our recent statewide survey, most rural obstetric providers in Nebraska confirmed that they already acquire these axial sweeps during routine care (14).

However, the diagnostic utility of this data remains limited due to a lack of infrastructure for standardized interpretation, clinical integration, and specialist referral pathways—gaps that AI can help fill. This is particularly important in rural and underserved areas where fetal cardiology expertise and high-quality image interpretation are often unavailable. Embedding AI into existing workflows can empower frontline providers to flag potential anomalies in real time, enabling consistent screening performance regardless of geographic location.

### Framework of proposed AI-driven approach

Our proposed framework leverages existing infrastructure and workflows to enhance prenatal detection of CHD without placing additional burden on frontline providers. Specifically, this initiative will involve community obstetricians across rural Nebraska and the Midwest, utilizing standard-of-care obstetric ultrasounds with no added technology or staffing requirements at the point of care. The goal is to seamlessly embed AI-based screening into current prenatal workflows, empowering providers to flag high-risk cases early without disrupting routine care delivery.

The foundation for this approach is a secure, cloud-based platform already in use for image sharing and teleconsultation. Community obstetricians will continue performing fetal anatomy surveys as usual; however, ultrasound images will now be automatically uploaded to this centralized platform, where trained AI algorithms will analyze fetal cardiac structures. We will train the AI model to recognize sonographic patterns and features associated with an elevated likelihood of CHD, specifically focusing on critical CCHD that benefits most from early diagnosis and coordinated delivery planning.

Once the AI flags a case as potentially high-risk, the system will generate a referral pathway to the nearest regional specialty clinic equipped with fetal cardiology expertise. These are not centralized referrals to a distant tertiary hospital; instead reflect a regionalized model of care that brings specialized services closer to the patient. We aim to decentralize fetal cardiac care by utilizing the existing network of Children's Nebraska cardiac outreach clinics with fetal imaging capabilities throughout the state and surrounding areas.

At these regional sites, fetal cardiologists and imaging specialists will perform comprehensive diagnostic testing, including fetal echocardiography, to confirm or rule out the presence of CHD. From there, mothers will receive appropriate counseling and follow-up, including delivery planning at specialized centers when needed. This AI-enabled model enables efficient triaging, ensures equitable access to advanced diagnostics, and ultimately bridges the geographic gap in prenatal CHD detection, especially for rural and underserved populations.

### Establishment of multi-disciplinary team

Team Members and Their Roles

1. Dr. Aaron Lanik

Title: Rural Residency Training Program Director and Associate Professor, UNMC Department of Family Medicine.

Expertise:

• Extensive experience in rural healthcare systems and addressing systemic barriers to care.
• Expertise in developing and implementing training programs for rural healthcare providers.

**Figure 4:**
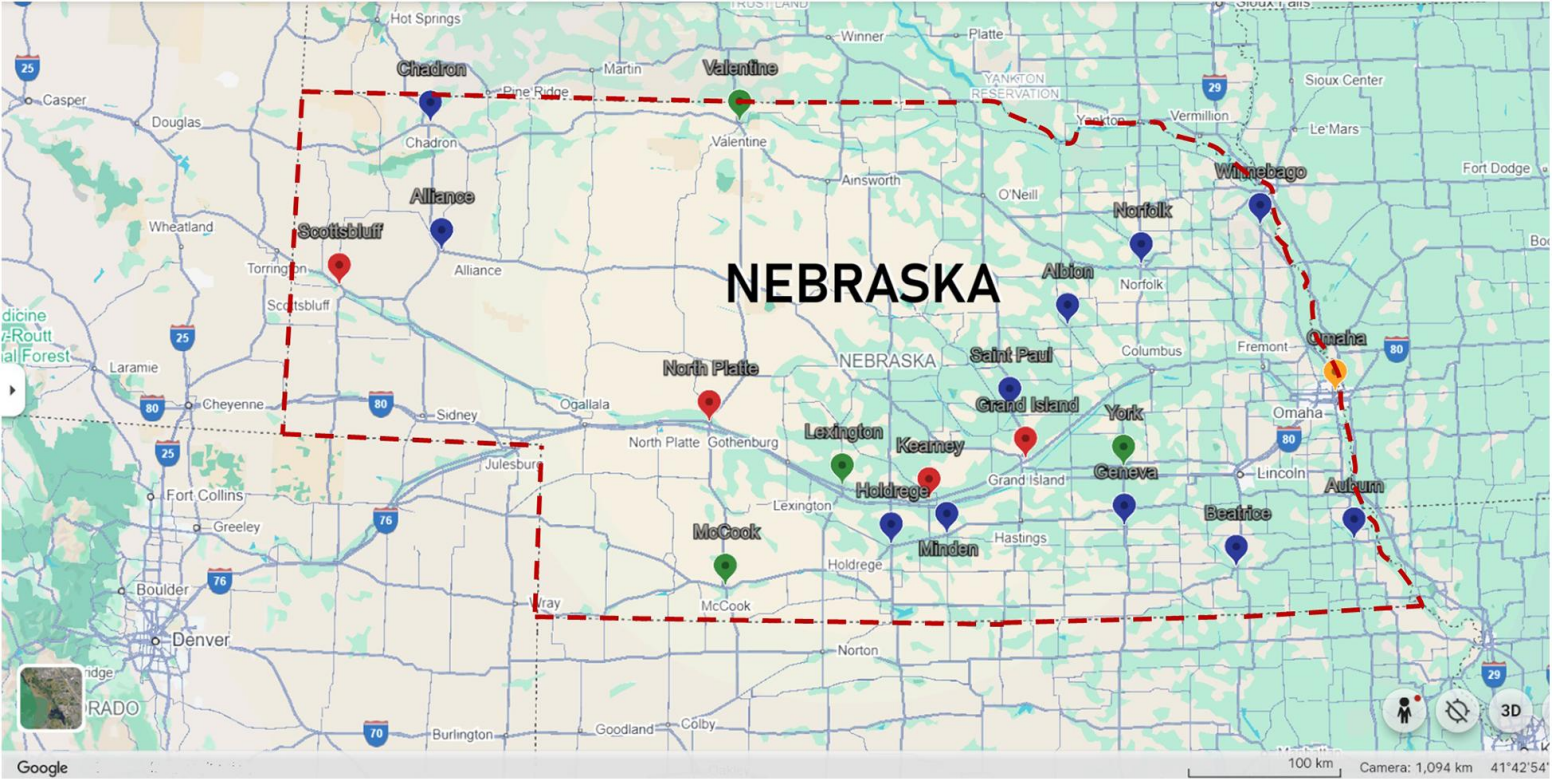
Map of university of Nebraska medical center (UNMC) family medicine rural residency program partnerships. Yellow pin indicates Family Medicine Rural Residency Program central site. Red pins indicate sites where Family Medicine Rural Residents complete the final two years of their training. Green pins represent locations where UNMC Family Medicine Residents complete 1–2 month rural rotations. Blue pins denote recent Family Medicine Rural Education sites.

Role in the Project:

• Facilitate partnerships with rural healthcare facilities to integrate AI-enhanced prenatal CHD screening programs, leveraging established rural clinical education partnerships (Figure 4).
• Provide insights into the unique healthcare challenges faced by rural populations, ensuring that solutions are specifically tailored to these settings.
• Lead the development of educational initiatives for healthcare providers to utilize AI technologies effectively.

2. Dr. Alex Foy

Title: Fetal and Pediatric Cardiologist, Assistant Professor of Pediatric Cardiology, UNMC Department of Pediatrics.

Expertise:

• Specializes in fetal and pediatric cardiology, with a focus on prenatal CHD detection and management.
• Experience with telehealth and tele-ultrasound technologies for remote diagnostic services.

Role in the Project:

• Collaborate on the design and implementation of AI models tailored to prenatal CHD detection.
• Provide clinical validation and interpretation of AI-detected anomalies to ensure diagnostic accuracy.
• Lead the development of regionalized outreach centers equipped with telehealth capabilities for confirmatory testing and follow-up care.

3. Dr. Jieqiong Wang

Title: Machine Learning Expert, Assistant Professor at UNMC

Expertise:

• Extensive experience in developing machine learning algorithms for medical applications.
• Expertise in integrating large-scale data and creating predictive models for clinical decision-making.

Role in the Project:

• Design and optimize AI/ML algorithms to improve prenatal CHD detection from ultrasound imaging data.
• Collaborate with imaging scientists and clinicians to ensure AI models are interpretable and clinically relevant.
• Lead efforts to train AI models on diverse datasets to improve generalization and diagnostic accuracy across different populations and healthcare settings.

4. Dr. Ling Li

Title: Imaging Scientist and Assistant Professor of Pediatric Cardiology, UNMC Department of Pediatrics.

Expertise:

• Advanced knowledge in AI and machine learning applications in medical imaging.
• Expertise in optimizing imaging technologies for enhanced diagnostic accuracy.

Role in the Project:

• Develop and implement AI models for CHD detection from routine prenatal ultrasound scans.
• Enhance the imaging-sharing platform to enable seamless data exchange and analysis across rural healthcare facilities.
• Conduct data analysis to evaluate the performance of AI models and ensure compliance with ethical and regulatory standards.

5. Dr. Jason Christensen

Title: Associate Professor of Pediatric Cardiology, UNMC; Director of Noninvasive Imaging

Expertise:

• Advanced multimodality cardiac imaging and machine learning.
• Translational imaging research and workflow optimization.

Role in the Project:

• Lead integration of AI into noninvasive imaging pathways.
• Support model refinement with clinical imaging data.
• Bridge diagnostic innovation with care delivery through AI-enhanced workflows.

6. Dr. Neil Hamill

Title: Associate Professor, UNMC Department of Obstetrics and Gynecology; Maternal-Fetal Medicine Physician, Nebraska Medicine

Expertise:

• Prenatal care and high-risk obstetrics.
• Clinical ultrasound and maternal-fetal imaging.

Role in the Project:

• Guide image quality assurance and AI model relevance for obstetric use.
• Support adoption of AI screening in routine maternal-fetal care.
• Collaborate on the development of training tools for obstetric ultrasound practitioners.

### Collaborative approach

The interdisciplinary collaboration will be established within the framework of the Criss Heart Center, currently led by Jeffrey W. Delaney, MD, Medical Director and Chief of Pediatric Cardiology and Ram Kumar Subramanyan, MD, PhD, Surgical Director and Chief of Pediatric Cardiothoracic Surgery. The project brings together a diverse and highly specialized team whose combined expertise addresses the clinical, technological, and systemic barriers to prenatal CHD detection and management. The collaboration spans rural health systems, fetal cardiology, maternal-fetal medicine, machine learning, diagnostic imaging, and clinical education.

## Discussion

This position paper presents a forward-looking, AI-enabled framework to improve prenatal screening for congenital heart disease (CHD), with a particular focus on addressing disparities in rural and underserved settings. Our retrospective review of 1,502 pediatric CHD surgical cases revealed a prenatal detection rate of only 36.7% overall and 32.7% in rural patients, underscoring a persistent and actionable gap in early diagnosis. These findings are consistent with prior reports showing lower prenatal detection rates in non-urban settings and highlight the need for novel solutions that can be integrated into existing clinical infrastructure (15).

Our proposed framework builds upon standard prenatal imaging practices, particularly the routine acquisition of fetal axial cine sweeps, which are commonly obtained in both urban and rural settings. By applying AI to these widely available imaging data, we aim to enhance early CHD detection without placing additional burden on frontline providers. Importantly, this approach is not theoretical; prior studies have demonstrated the feasibility and diagnostic accuracy of machine learning (ML) models in detecting a range of fetal anomalies (16), including cardiac defects (12), fetal renal anomalies (17), and neural tube defects (18). These advances establish a strong foundation for expanding AI applications into routine prenatal screening, particularly in environments where access to pediatric cardiology expertise is limited.

However, the successful implementation of this framework requires careful consideration of both technical and ethical challenges (19, 20). Variability in ultrasound image quality, acquisition technique, and equipment across clinical sites can affect model performance (21, 22). To mitigate this, model training must incorporate diverse, multicenter/site datasets that reflect real-world heterogeneity (22, 23). Generalizability must also be rigorously tested to ensure consistent performance across various patient populations, gestational ages, and care settings (22, 23). Equally critical is the need to address potential algorithmic bias, particularly racial, geographic, or socioeconomic bias, that could inadvertently reinforce existing disparities if not carefully validated (19, 24).

From an ethical standpoint, transparency, explainability, and clinician oversight are essential. AI outputs must be interpretable and integrated into clinical workflows in a manner that supports, rather than replaces, professional judgment (23). It is essential that both patients and providers have confidence in the rationale behind AI-generated referrals, especially when these decisions may influence critical factors such as delivery planning or care location (23).

Additional barriers to AI adoption include provider acceptance, infrastructure variability, and reimbursement concerns (25–27). Rural clinics may lack access to high-speed internet, sufficient staff capacity, or familiarity with AI-based tools. Therefore, the sustainability of the proposed model depends on integrating it into existing telehealth platforms, offering user-friendly interfaces, and ensuring that referral networks are supported through regional partnerships, such as those already established with Children's Nebraska. Clinician training will also be essential, not only to interpret AI outputs, but to build trust and facilitate uptake. Educational programs focused on AI literacy, sonographic standards, and feedback mechanisms will support long-term adoption.

## Conclusion

There is a crucial need to improve prenatal detection rates of CHD. The integration of AI technologies into prenatal CHD detection represents a transformative step forward in addressing healthcare disparities in rural Nebraska and the Midwest. Through the unique and complementary expertise of our multi-disciplinary team, we submit that this approach has the potential to set a national precedent for leveraging AI to enhance maternal-fetal health in resource-limited settings. We believe that collaborative efforts with healthcare institutions, technology developers, and community stakeholders will ensure the project's success and long-term sustainability.

## Data Availability

The original contributions presented in the study are included in the article/Supplementary Material, further inquiries can be directed to the corresponding author.
